# Sternal reconstruction for unusual chondrosarcoma: innovative technique

**DOI:** 10.1186/1749-8090-7-40

**Published:** 2012-05-02

**Authors:** Mario Nosotti, Lorenzo Rosso, Paolo Mendogni, Davide Tosi, Alessandro Palleschi, Antonina Parafioriti, Luigi Santambrogio

**Affiliations:** 1 Thoracic Surgery and Lung Transplantation Unit, Fondazione IRCCS Cà Granda Ospedale Maggiore Policlinico, University of Milan, Via Francesco Sforza, 35, Milan, 20122, Italy; 2 Pathology Department, Orthopaedic Institute Gaetano Pini, Milan, Italy

**Keywords:** Chondrosarcoma, Sternum, Transplantation

## Abstract

The authors report a clinical case of a primary sternal chondrosarcoma, presented as a mass in the anterior mediastinum. The patient was treated with subtotal sternectomy and sternal transplantation followed by radiotherapy. Twelve months after surgery, the patient is in good clinical condition, without any sign of tumor relapse and with normal respiratory mechanics.

Primary malignant tumors of the sternum are uncommon and a presentation mimicking thymoma is rare and unreported. The stermal replacement with a cryopreserved allograft sternum is an innovative technique that overcomes the problems related to the prosthetic biocompatibility or to the bone autograft.

## Background

Primary malignant tumors of the chest wall are uncommon; nevertheless, chondrosarcoma is the most common malignant tumor of the bony thorax and the single most common malignancy of the sternum. The authors report a successful surgery for primary sternal chondrosarcoma, which was treated with subtotal sternectomy and sternal transplantation.

## Case presentation

**Figure 1:**
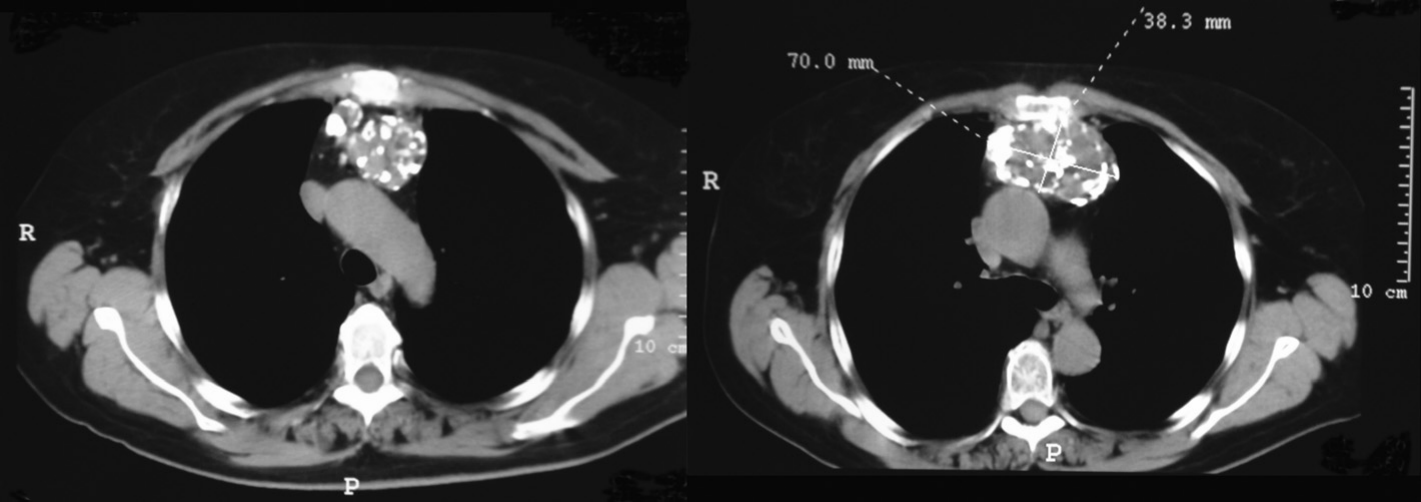
**CT images show the mass into the anterior mediastinum.** On the right it is possible to observe the calcified connection between the mass and the sternum; no cortical erosion is present.

A 71-year-old female was referred to our Unit for anterior thoracic pain associated with a mass of the anterior mediastinum. The CT-scan detected a calcified shadow measuring 10 × 7 cm that suggested a thymoma (Figure 1). The lesion was immediately adjacent to the posterior sternal profile without any erosion of the cortex; on the contrary, one of the calcifications appeared to fusion to the left posterior sternal margin. The PET-scan was silent except for a small focal spot between the mediastinal mass and the right posterior sternal margin, corresponding approximately to the referred pain. The clinical examinations for myasthenia gravis (physical examination, antibodies against the acetylcholine receptor and single fiber electromyography) were negative. We avoided a biopsy of the lesion, considering that the tumors of the anterior mediastinum suspected for thymoma are directly referred to surgery. Keeping in mind the local fusion of the “thymoma” with the sternum and the PET spot suspected for a focal malignancy, cryopreserved allograft sternum and costal cartilage were prepared by the Regional Bank for Muscle and Bone Tissue (Banca Regionale di Tessuto Muscolo-Scheletrico, Istituto Ortopedico G. Pini, Milano, trapianti@gpini.it) in the case of sternal resection.

**Figure 2:**
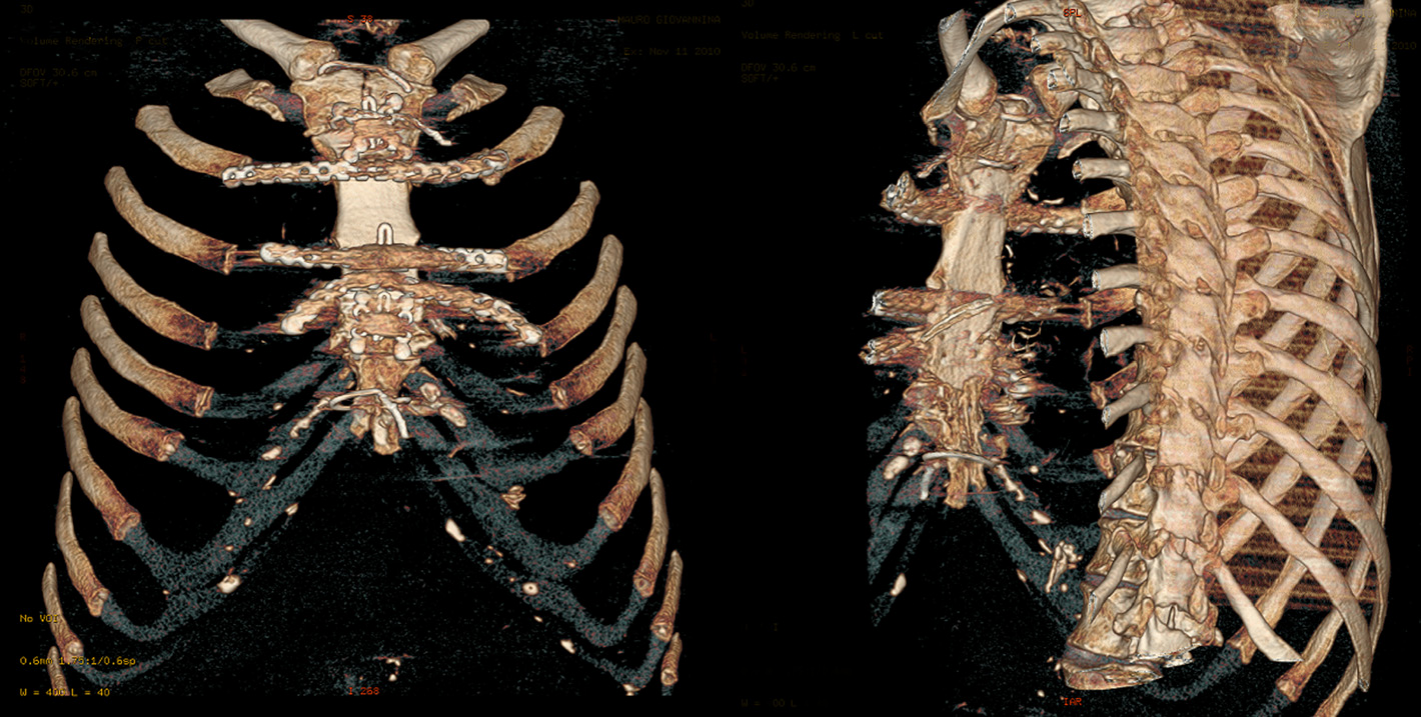
**CT scan reconstruction 2 months after surgery.** The transplanted sternum appears well integrated with the rib cage.

The firm adhesion of the tumor with the sternal body became evident at the sternotomy. A frozen section suspected a tumor of cartilage; consequently, the mediastinal mass, the sternal body and the costal cartilages were resected en bloc. For reconstruction we used the cryopreserved allograft properly tailored and fixed to the sternal manubrium and to the ribs. Titanium plates bended on site were used to fix the graft (Synthes, Oberdorf, Switzerland). The chest wall stability was excellent and the postoperative course was uneventful (Figure 2). Final histology was positive for chondrosarcoma. The residual native sternum stumps received adjuvant radiotherapy. Twelve months after surgery the patient is in good clinical condition, without any sign of tumor relapse; in addition, the thoracic wall is stable with normal respiratory movements.

## Conclusions

Primary malignant tumors of the sternum are really rare, and account approximately for 0.5% of primary bone tumors. The chondrosarcoma is the most common primary malignant tumor of the sternum. This tumor generally occurs in young adult men; the patient is usually presented with a gradually growing, painful, hard and fixed mass of the sternum. On chest radiograph, chondrosarcoma commonly appears as a lobulated mass originating from the medullary portion of the bone, the margin is not well defined and the destruction of cortex is common. The chondrosarcoma is generally radiolucent but stippled, ring-like or arc-like calcifications are usually present [1].

To the authors’ knowledge, their case is the first report of a chondrosarcoma originating from the posterior face of the sternum, integrally preserving the cortex and growing in the anterior mediastinum like a thymoma.

Wide excision remains the key for good local control; total sternectomy must be carried out if the sternum is entirely involved, but a subtotal resection, if possible, is recommended in order to partially preserve the chest wall stability [2]. When a portion of soft tissue must be resected, flap or skin grafting procedures can be utilized. Reconstruction of a large defect needs prosthetic material such as prolene mesh, metal implants, methyl-methacrylate bone cement, polyethylene, etcetera [3]. Unfortunately, ideal prosthetic material does not exist; excessive rigidity can erode adjacent structures, rejection or infection may occur and a real integration of the foreign material is quite impossible.

Following the theory of regenerative medicine, we believe bone graft is the best substitute for the resected sternum; the graft could act as a scaffold for new bone formation, mesenchymal cells could migrate into the graft and a differentiation into osteoblastic elements has been demonstrated [4]. In the case of a large graft, as sternal substitution requires, we prefer allografts to autografts in order to avoid pain and functional impairment on the donor site. Moreover, the cryopreserved bone graft is free from any immunogenic capacity.

From a practical point of view, the cryopreserved sternum is easily manipulated and tailored: a perfect fitting of the osteochondral graft into the defect could be achieved in a few minutes. Time consuming is the fixation of the graft by screws and plates, notwithstanding, the final impression is a satisfactory picture of a solid and biocompatible work.

The sternal replacement with a cryopreserved allograft is a new procedure needing validation; our report follows the first by the Padua group [5], but considering such a technique promising and rare, we stress the opportunity to accumulate the efforts of all surgical centers into an international file.

## Consent

Written informed consent was obtained from the patient for publication of this case report and accompanying images. A copy of written consent is avaiable for review by the Editor-in-Chief of this journal.

## Competing interests

The authors declare that they have no competing interests.

## Authors’ contribution

MN, LR and LS performed surgical operation, designed research and analyzed data. PM, DT and AP performed research and collected data. AP analyzed data and results. MN wrote the article. All Authors read and approved the final manuscript.

## References

[B1] Somers J Faber LP Chondroma and chondrosarcoma Semin Thorac Cardiovasc Surg 1999 11 270 277 10451259 10.1016/s1043-0679(99)70068-7

[B2] Chapelier AR Missana MC Couturaud B Sternal resection and reconstruction for primary malignant tumors Ann Thorac Surg 2004 77 1001 1006 10.1016/j.athoracsur.2003.08.053 14992915

[B3] Ashford RU Stanton J Khan F Pringle JA Cannon SR Briggs TW Surgical treatment of chondrosarcoma of the sternum Sarcoma 2001 5 209 213 10.1080/13577140120099209 18521316 PMC2395459

[B4] Granero-Molto F Weis JA Longobardi L Spagnoli A Role of mesenchymal stem cells in regenerative medicine: application to bone and cartilage repair Expert Opin Biol Ther 2008 8 255 268 10.1517/14712598.8.3.255 18294098

[B5] Marulli G Hamad AM Cogliati E Breda C Zuin A Rea F Allograft sternochondral replacement after resection of large sternal chondrosarcoma J Thorac Cardiovasc Surg 2010 139 e69 e70 10.1016/j.jtcvs.2009.01.007 19660292

